# Innovations in the national household random sampling in Brazilian National Health Survey: results from Starfield and Shi’s adult primary care assessment tool (PCAT)

**DOI:** 10.1186/s12939-021-01455-w

**Published:** 2021-05-01

**Authors:** Luiz Felipe Pinto, Otavio Pereira D’Avila, Lisiane Hauser, Erno Harzheim

**Affiliations:** 1 Department of Medicine in Primary Health Care, Faculty of Medicine, Federal University of Rio de Janeiro (UFRJ), 36 Laura de Araújo Street, Cidade Nova, Rio de Janeiro, RJ 20551-031 Brazil; 2 Post-Graduate Studies Program in Epidemiology, Federal University of Rio Grande do Sul (UFRGS), 2400 Ramiro Barcellos Street, Santa Cecília, Porto Alegre, RS 90035-003 Brazil; 3 Federal University of Pelotas (UFPel), 457 Gonçalves Chaves Street, Centro, Pelotas, RS 96015-560 Brazil

**Keywords:** Househould surveys, PCAT, Evaluation of health systems, Primary care, Brazil, Inquéritos domiciliares, PCAT, Avaliação dos sistemas de saúde, Atenção primária à saúde, Brasil

## Abstract

**Background:**

Several middle and upper income countries carry out household surveys that seek to trace the profile of access and use of health services. Probably one of the most ambitious examples is Brazil, with its National Health Survey (PNS-2019). We evaluated PNS-2019, presenting in an unprecedented way, one of its innovations, which refer to Starfield and Shi’s adult Primary Care Assessment Tool (PCAT).

**Methods:**

Based on a cross-sectional study, we evaluated Module H of the PNS-2019, which interviewed a probabilistic sample of about 10,000 adults in 2019 in all 27 Brazilian states. According to the PCAT methodology, an average score equal to or above 6.6 indicates a greater orientation and quality of the evaluated primary care services.

**Results:**

Brazilian overall PCAT score [5, 9] reveals the need to improve primary health care services across the country. There were no statistically significant differences in the scores by sex (men and women, 5.9), and race (whites 5.9 [5.7; 6.0] and brown / black 5.9 [5.8; 6.0]). On the other hand, there was a difference in terms of age. The elderly evaluated the services in a more positive way (score = 6.1 [6.0; 6.2]), when compared to those aged 40–59 years (5.9 [5.7; 6.0]) and 18 to 39 years (5.6 [5.5; 5.8]). First results of PNS-2019 show that the population that most needs primary care services in SUS is the one with the best perception and the most positive evaluation of the actions and procedures offered in health facilities.

**Discussion:**

During 2019, Brazil undertook important structural reforms in PHC based on a new financing model with the aim of inducing an improvement in efficiency and strengthening its attributes. It is essential that countries with universal health coverage (UHC) guarantee access to their population and, especially, the most vulnerable, seek better efficiency of these services and regularly assess PHC based on the population’s perception, through an independent methodology that monitor the quality of services and the strength of PHC, generating value for public resources applied to health services.

## Resumo

**Introdução:**

Vários países de renda média e alta realizam pesquisas domiciliares que procuram traçar o perfil de acesso e uso dos serviços de saúde. Provavelmente um dos exemplos mais ambiciosos é o Brasil, com sua PNS. Avaliamos esse inquérito e apresentamos, de forma inédita uma de suas inovações, que se refere ao uso do instrumento Primary Care Assessment Tool (PCAT).

**Métodos:**

Com base em um estudo transversal, avaliamos o Módulo H da PNS-2019, que entrevistou uma amostra probabilística de cerca de 10.000 adultos em 2019 em todos os 27 estados brasileiros. De acordo com a metodologia do PCAT, um escore médio igual ou acima de 6,6 indica uma maior orientação e qualidade dos serviços de atenção primária avaliados.

**Resultados:**

O escore médio geral do PCAT no Brasil foi de 5,9, revelando a necessidade de melhoria dos serviços de atenção primária à saúde em todo o país. Não houve diferenças estatisticamente significantes nos escores por sexo (homens e mulheres, 5,9) e raça (brancos 5,9 [5,7; 6,0] e pardos / pretos 5,9 [5,8; 6,0]). Por outro lado, houve diferença em termos de idade. Os idosos avaliaram os serviços de forma mais positiva (escore = 6,1 [6,0; 6,2]), quando comparados aos de 40–59 anos (5,9 [5,7; 6,0]) e 18 a 39 anos (5,6 [5,5; 5,8]). Os primeiros resultados da PNS-2019 mostram que a população que mais necessita dos serviços de atenção básica no SUS é aquela que tem melhor percepção e avaliação mais positiva das ações e procedimentos oferecidos nas unidades de saúde.

**Discussão:**

Durante o ano de 2019, o Brasil fez reformas estruturais importantes na APS a partir de um novo modelo de financiamento, com o objetivo de induzir uma melhoria da eficiência e fortalecer seus atributos. É fundamental que países com sistemas de saúde de cobertura universal, garantam acesso a sua população e, em especial, aos mais vulneráveis; busquem melhor eficiência desses serviços e avaliem regularmente a APS a partir da percepção da população, por meio de uma metodologia independente que monitore a qualidade dos serviços e a força da APS, gerando valor aos recursos públicos aplicados nos serviços de saúde.

## Introduction

Brazil is a country of continental dimensions with a population of 212 million inhabitants, formed by five major regions, twenty-seven units of the federation, and more than 5500 municipalities; autonomous administrative entities that together contribute to forming the so-called Unified Health System (SUS). Over the past few decades, several middle and upper income countries carry out household surveys that seek to trace the profile of access and use of health services [1–4]. Among the largest economies in the world, one of the most ambitious examples is Brazil, which regularly conducts the National Health Survey (PNS) with modules that reproduce internationally validated instruments and aim to assess the health situation of the Brazilian population. In the Brazilian Unified Health System (SUS), primary health care (PHC) is mainly developed in health centers or family health units, formed essentially by the so-called Family Health teams (eSF), composed of family physician, a PHC nurse, a nursing technician, a community health agent, and in various services, by a health surveillance agent. Until July 2020, the Ministry of Health registered a total of 43,639 eSF in all 27 states of the country [5]. The challenge of evaluating the performance of this system in a continental country such as Brazil and comparing regional and local realities, makes it necessary to establish new inter-institutional partnerships and the use of standardized and statistically validated instruments. According to Harzheim et al. [6], published by the IJEH in late 2019 (10.1186/s12939-019-1083-2), the challenge of evaluating primary health care was launched in August 2019 by the Institute Brazilian Geography and Statistics (IBGE), the Brazilian National Census Bureau. IBGE innovated by including a question module to assess the quality of primary health care services in its main national household survey (PNS-2019).

## Brazilian pioneering spirit and innovation in the use of PCAT

We present to the scientific community around the world, results of this assessment, launched by IBGE on October 21, 2020. To our knowledge, it is unprecedented for a national statistical institute in a country to use probabilistic and representative samples of capitals, metropolitan regions, cities in the inner, all Brazilian states and five regions to draw a picture of the quality of PHC services from the perspective of adults who use basic health facilities or family health units. Interviewing around 10,000 people, the methodology used was one of the versions of the instrument validated internationally and published in Brazil by the Ministry of Health in 2010 and updated in 2020 [7], entitled “Primary Care Assessement Tool” (PCATool). From the short version with 25 questions [8] (Module H of PNS-2019) that include items of all attributes defined by Starfield and Shi, it was possible to objectively measure the degree of orientation of services to PHC. We are referring to the general synthesis score (overall score), obtained by calculating the average of the responses of adults, transformed into a score of (0–10). The authors defined the value of 6.6 as the cutoff point, that is, services oriented to primary care should have at least this score to be considered of quality.

## Main results

Also following the PCAT methodology, adults who evaluated services were those who had at least two appointments with the same doctor in the last 6 months at the same heatlh facility. As it is a survey with a probabilistic sample, IBGE calculated the so-called “sample expansion factors”, which, taken together, now represent a universe of 17.3 million people (that is, each person in the sample who answered the instrument represented, on average, in 1730 in the population). Of this total, 69.9% were women, 60.9% of people were black or brown; 65.0% had spouses; 35.8%, 40 to 59 years old [9] .

Results obtained in PNS-2019 are slightly below the value of 6.6 as the PCAT cutoff escore point: 5.9, with no statistically significant differences between sex (men and women, score = 5.9) and race (white score = 5.9 [CI: 5.7; 6.0] and brown / black score = 5.9 [CI: 5.8; 6.0]). On the other hand, differences between the age groups stood out: the elderly evaluated the services in a more positive way (score = 6.1 [CI: 6.0; 6.2]), when compared to adults aged 40–59 years (score = 5.9 [CI: 5.7; 6.0]) and those aged 18 to 39 years (score = 5.6 [CI: 5.5; 5.8]). These same elderly people, who are usually the ones who most frequent health units, and, also the population with some morbidity, were the ones who best evaluated the services. For example, hypertensive (score = 6.2), diabetic (score = 6.3), cardiac patients (score = 6.4), asthmatics (score = 6.0), people with chronic lung diseases (score = 6.4) and depression (score = 6.1). Also, a better performance was observed among: [1] those who received at least one home visit from a community agent or another member of the family health team (score = 6.1), when compared to those who never received a visit (score = 5.7), [2] those who were visited by a health surveillance agent (score = 6.0), in contrast to those who were never visited (score = 5.6), [3] those who are registered at a family health unit (score = 6.0) vs who was not registered (score = 5.5).

## Conclusion

Brazil has faced an economic crisis since 2016 (even before the COVID-19 pandemic). First results of PNS-2019 show that the population that most needs primary care services in SUS is the one with the best perception and the most positive evaluation of the actions and procedures offered in health facilities. Still, PHC services have shown a low PHC orientation (overall brazilian PCAT score < 6,6). During 2019, Brazil undertook important structural reforms in PHC based on a new financing model [10] with the aim of inducing an improvement in efficiency and strengthening its attributes. It is essential that countries with universal health coverage (UHC) guarantee access to their population and, especially, the most vulnerable, seek better efficiency of these services and regularly assess PHC based on the population’s perception, through an independent methodology that monitor the quality of services and the strength of PHC, generating value for public resources applied to health services. The history of western countries in primary health care assessment can be divided over the twenty-first century into two moments: before and after the contributions of the team led by Professors Barbara Starfield and Leiyu Shi of Johns Hopkins School of Public Health in Baltimore, Maryland, United States of America. And this methodology was left as a legacy by the brilliant Professor Barbara Starfield, being more disseminated by her great partner, Professor Leiyu Shi.

Brazilian National Health Survey (PNS-2019) demonstrated that it is possible to use an instrument of evaluation of primary care services with rigor and statistical representativeness, making its results comparable between several countries of the world and serving as a baseline for the definition of policies public access and universal health coverage.

## Data Availability

The National Survey of Health (PNS-2019) was launched on October 21st, 2020 and availability of data is on IBGE homepage: https://ftp.ibge.gov.br/PNS/2019/Divulgacoes/Volume_2/tabelas_xls/Modulo_H.zip

## Abbreviations

eSF: Equipe de Saúde da Família.
IBGE: Instituto Brasileiro de Geografia e Estatística (Brazilian Census Bureau)
PCAT: Primary Care Assessment Tool
PHC: Primary Health Care
PNS: Pesquisa Nacional de Saúde (Brazilian National Health Survey)
SUS: Sistema Único de Saúde (Brazilian Unified Health System)
UHC: Universal Health Coverage
